# A Multi-Method Survey on the Use of Sentiment Analysis in Multivariate Financial Time Series Forecasting

**DOI:** 10.3390/e23121603

**Published:** 2021-11-29

**Authors:** Charalampos M. Liapis, Aikaterini Karanikola, Sotiris Kotsiantis

**Affiliations:** Department of Mathematics, University of Patras, 26504 Patras, Greece; sotos@math.upatras.gr

**Keywords:** time series forecasting, machine learning, financial time series, sentiment analysis, FinBERT, multivariate, multistep, regression, Twitter

## Abstract

In practice, time series forecasting involves the creation of models that generalize data from past values and produce future predictions. Moreover, regarding financial time series forecasting, it can be assumed that the procedure involves phenomena partly shaped by the social environment. Thus, the present work is concerned with the study of the use of sentiment analysis methods in data extracted from social networks and their utilization in multivariate prediction architectures that involve financial data. Through an extensive experimental process, 22 different input setups using such extracted information were tested, over a total of 16 different datasets, under the schemes of 27 different algorithms. The comparisons were structured under two case studies. The first concerns possible improvements in the performance of the forecasts in light of the use of sentiment analysis systems in time series forecasting. The second, having as a framework all the possible versions of the above configuration, concerns the selection of the methods that perform best. The results, as presented by various illustrations, indicate, on the one hand, the conditional improvement of predictability after the use of specific sentiment setups in long-term forecasts and, on the other, a universal predominance of long short-term memory architectures.

## 1. Introduction

The observation of the evolution of various time-dependent phenomena, as well as the decision-making based on structures predicting their future behavior have greatly shaped the course of human history. The emergence of the need of the human species for knowledge of the possible future outcomes of various events could only lead to the development and use of methods aimed at extracting reliable predictions.Their success, however, is not necessarily inferred from the emergence of need.The research field of predicting sequential and time-dependent phenomena is called *time series forecasting*.

Specifically, time series forecasting is the process in which the future values of a variable describing features of a phenomenon are predicted based on existing historical data using a specific fit abstraction, i.e., a model. All such time-dependent features containing past observations are represented as time series. The latter then constitute the input of each forecasting procedure. Time series are sequences of time-dependent observations extracted at specific time points used as their indexes. The sampling rate varies according to the requirements and the nature of the problem. In addition, depending on the number of attributes, i.e., the dependent variables describing observations recorded sequentially over the predefined time steps, whose values are collected at any given time, a distinction is made between univariate and multivariate time series [1]. Such methods find application in a wide range of time-evolving problems. Some examples include rainfall forecasts [2], gold [3] or stock price market predictions [4], as well as forecasting the evolution of epidemics such as the current COVID-19 pandemic [5,6]. The domain has flourished in recent decades, as the demand for better and better models remains increasingly urgent, as their use can greatly contribute to the optimization of decision-making and thus lead to better results in various areas of human interest.

In terms of forecasting procedures, during the first decades of development, methods derived from statistics dominated the field. This was based on the reasonable assumption that, given the nature of the problem, knowing the statistical characteristics of time series is the key to understanding their structure, and therefore predicting their future behavior. Currently, these methods—although still widely used—have been largely surpassed in performance by methods derived from the field of machine learning. Numerous such predictive schemes are based on regression models [7,8], while recently, deep-machine-learning architectures such as long short-term memory (LSTM) [9,10] are gaining ground. In addition, advances in natural language processing in conjunction with the fact that many time-dependent phenomena are influenced by public opinion lead to the hypothesis that the use of linguistic modeling containing information related to the phenomenon in question could improve the performance of forecasting procedures. Data containing relevant information is now easy to retrieve due to the rapid growth of the World Wide Web initially and social networks in recent years, and it is therefore reasonable to examine the utilization of such textual content in predictive schemes.

This work is a continuation of a previous comparative study of statistical methods for univariate time series forecasting [11], which now focuses on methods belonging to the category of machine learning. Comparisons involve results from an extended experimental procedure regarding mainly a wide range of multivariate-time-series-forecasting setups, which include sentiment scores, tested in the field of financial time series forecasting. Below, the presentation of the results is grouped as follows: Two distinct case studies were investigated, the first of which concerns the use of sentiment analysis in time series forecasting, while the second contains the comparison of different time-series-prediction methods, all of which were fit in datasets containing sentiment score representations. In each of these two scenarios, the evaluation of the results was performed by calculating six different metrics. Three forecast scenarios were implemented: single-day, seven-day, and fourteen-day forecasts, for each of which the results are presented separately.

## 2. Related Work

The field of time series forecasting constitutes—as already mentioned—a very active area of research. Growing demand for accurate forecasts has been consistently established over the last few decades for many real-world tasks. Various organizations, from companies and cooperatives to governments, frequently rely on the outcomes of forecasting models for their decisions to reduce risk and improvement. A constant pursuit of increasing predictive accuracy and robustness has led the scientific community in several different research directions. In this context, and provided there is a strong correlation between the views of individuals and the course of specific sequential and time-dependent phenomena, it is both reasonable and expected to approach such problems by intersecting the field of forecasting with that of opinion mining [12,13]. Thus, there are several approaches that focus on trying to integrate information extracted using sentiment analysis techniques in predictive scenarios. This section tracks the relevant literature, focusing on works that investigate the aforementioned approach.

Time-series-forecasting problems can be reduced to two broad categories. The first one consists of tasks in which the general future behavior of a time series must be predicted. Such problems can be considered classification problems. On the other hand, when the forecast outputs the specific future values that a time series is expected to take, then the whole process can be reframed as a regression task. Regarding the first class of problems, the relevant literature contains a number of quite interesting works. In [14], a novel method that estimates social attention to stocks by sentiment analysis and influence modeling was proposed to predict the movement of the financial market when the latter is formalized as a classification problem. Five well-known classifiers in Chinese stock data were used to test the efficiency of the method. For the same purpose, a traditional ARIMA model was used, together with information derived from the analysis of Twitter data [15], strongly suggesting that the exploitation of public opinion enhances the possibility of correctly predicting the rise or fall of stock markets. Similar results were achieved in [16], where the application of text-mining technology to quantify the unstructured data containing social media views on stock-related news into sentiment scores increased the performance of the logistic regression algorithm. A more sophisticated approach that employs deep sentiment analysis was used to improve the performance of an SVM-based method in [17], indicating once again that sentiment features have a beneficial effect on the prediction.

Predicting the actual future values of a time series, on the other hand, is a task far more difficult than predicting merely the direction of a time series. Therefore, there are a significant number of studies directed towards this research area as well. In [18], different text preprocessing strategies for correlating the sentiment scores from Twitter scraped textual data with Bitcoin prices during the COVID-19 pandemic were compared, to identify the optimum preprocessing strategy that would prompt machine learning prediction models to achieve better accuracy. Twitter data were also used in [19] to predict the future value of the SSECI (Shanghai Stock Exchange Composite Index) by applying a NARX time series model combined with a weighted sentiment representation extracted from tweets. In [20], given that the experimental procedure involved both data related only to a certain stock, as well as a small number of compared algorithms, sentiment analysis of RSS news feeds combined with the information of SENSEX points was used to improve the accuracy of stock market prediction, indicating that the use of the sentiment polarity improves the prediction.

As recent research work has indicated, given that there is a series of applications where deep-learning methods tend to perform better than either the traditional statistical [21] and the machine-learning-based ones [22], it is expected that such methods would also be used along with sentiment analysis techniques to achieve even greater accuracy in forecasting tasks. In [23], an improved LSTM model with an attention mechanism was used on AAPL (NASDAQ ticker symbol for Apple Inc) stock data, after adopting empirical modal decomposition (EMD) on complex sequences of stock price data, utilizing investors’ sentiment to forecast stocks, while in [24], the experimental procedure over six different datasets indicated that the fusion of network public opinion and realistic transaction data can significantly improve the performance of LSTMs. Both works demonstrated that the use of sentiment modeling improves the performance of LSTMs, but the amount of data used does not seem to be sufficient to substantiate a clear and general conclusion.

In addition, in several works [25,26] ensemble-based techniques have also been utilized together with sentiment analysis for time series forecasting in order to exploit the benefits of ensemble theory. In [27], an ensemble method, formed by combining LSTMs and ARIMA models under a feedforward neural network scheme, was proposed in order to predict future values of stock prices, utilizing sentiment analysis on data provided by scraping news related to the stock from the Internet. Moreover, an ensemble scheme that combines two well-known machine-learning algorithms, namely support vector machine (SVM) and random forest, utilizing information related to the public’s opinion about certain companies by incorporating sentiment analysis by the use of a trained word2vec model was proposed in [28]. Despite the results taken from the experimental procedure indicating that there were cases in which the ensemble model performed better than its constituents, the overall performance of the model depended on both the volume and the nature of the data available.

In terms of extended studies that focus on the extensive comparison of several different methods, given that multiple sentiment analysis schemes are also incorporated to predict the future values of time series, to our knowledge, only a relatively more limited number of works seem to exist in the current literature. Some of them are listed below. Various traditional ML algorithms, as well as LSTM architectures were tested over financial data by exploiting the use of sentiment analysis on Twitter data in [29], while a survey of articles that focused on methods that touch up the predictions of stock market time series using financial news from Twitter, along with a discussion regarding the improvement of their performance by speeding up the computation, can be found in [30]. Given the above, the present work aspires to constitute a credible insight into the subject, specifically regarding the behavior of a large number of forecasting methods in light of their integration with sentiment analysis techniques.

## 3. Experimental Procedure

In the extensive series of experiments performed, a total of 27 algorithms were tested for their performance in relation to a corresponding multivariate dataset consisting, on the one hand, of the time series containing the daily closing values of each stock as a fixed input component and, on the other, of one of a plurality of 22 different sentiment score setups. A total of 16 initial datasets of stocks containing such closing price values from a period of three years, starting from 2 January 2018 to 24 December 2020, were used. Three different sentiment analysis methods were utilized to generate sentiment scores from linked textual data extracted from the Twitter microblogging platform. Moreover, a seven-day rolling mean strategy was applied to the sentiment scores, leading to six distinct time-dependent features. A number of 22 combinations, per algorithm, of distinct input components, from the calculated sentiment scores together with the closing values, were tested under the multivariate forecasting scheme. Thus, given the aforementioned number of features and setups, a total of 16datasets×22combinations×27algorithm×3shifts= 28,512 experiments were performed.

### 3.1. Datasets

As already mentioned, 16 different initial datasets containing the time series of the closing values of sixteen well-known listed companies were used. All sets include data from the aforementioned three-year period, meaning dates starting from 2 January 2018 to 24 December 2020. Table 1 shows the names and abbreviations of all the shares used.

**Figure 1:**
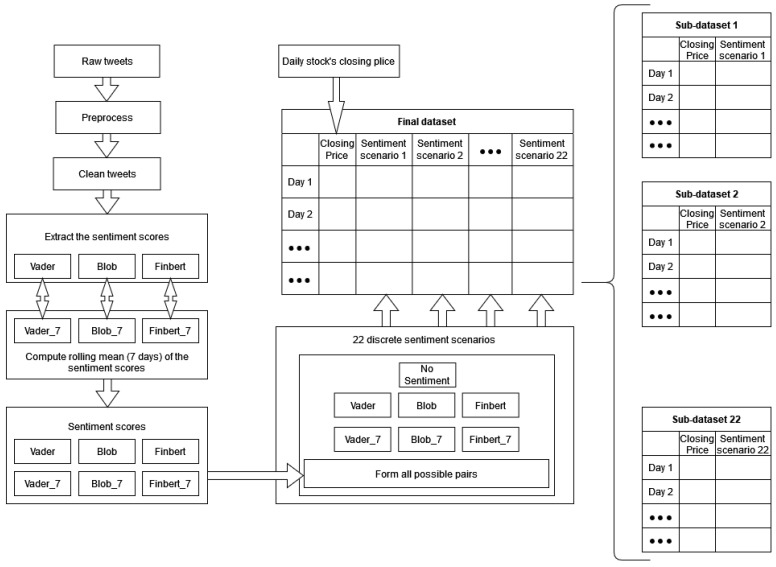
Final datasets’ construction process.

However, each of the above time series containing the closing prices of the shares was only one of the features of the final multivariate dataset. For each share, the final datasets were composed by introducing features derived from a sentiment analysis process, which was applied to an extended corpus of tweets related to each such stock. Figure 1 depicts a representation of the whole process, from data collection to the creation of the final sets. Below is a brief description of each stage of the final-dataset-construction process.

#### 3.1.1. Raw Textual Data

First, a large number of—per stock—related posts were collected from Twitter and grouped per day. These text data include tweets written exclusively in English. Specifically, the tweets were downloaded using the Twitter Intelligence Tool (TWINT) [31], an easy-to-use Python-based Twitter scraper. TWINT is an advanced, standalone, yet relatively straightforward tool for downloading data from user profiles. With this tool, a thorough search for stock-related reports to be investigated—that is, tweets that were directly or indirectly linked to the share under consideration—resulted in a rather extensive body of text data, consisting of day-to-day views or attitudes towards stocks of interest. These collections were then preprocessed and moved to the sentiment quantification extraction modules.

#### 3.1.2. Text Preprocessing

**Figure 2:**
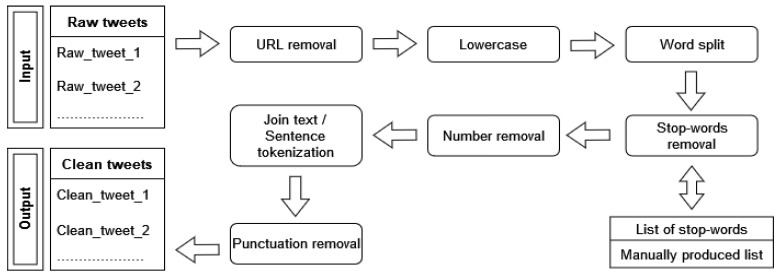
Text-preprocessing scheme.

Next, the text-preprocessing step schematically presented in Figure 2 followed. Specifically, after the initial removal of irrelevant hyperlinks and URLs, using the *re* Python library [32], each tweet was converted to lowercase and split into words. A series of numerical strings and terms of no interest taken from a manually created set was then removed. Lastly, on the one hand—and after the necessary joins to bring each text to its initial structure—each tweet was tokenized according to its sentences using the *NLTK* [33,34] library, and on the other, using the *string* [35] module, targeted punctuation removal was applied.

#### 3.1.3. Sentiment Scores

The next step involved generating the sentiment scores from the collected tweets. In this work, three distinct sentiment analysis methods, that is the sentiment modules from *TextBlob* [36], the *Vader* [37] Sentiment Analysis tool, and *FinBERT* [38], a financial-based fine-tuning of the *BERT* [39] language representation model, were used. For each of the above, and given the day-to-day sentiment scores extracted with the use of each one of them, a daily mean value formed the final collection of sequential and time-dependent instances that constituted the sentiment-valued time series of every corresponding method. It should be noted that, in addition to the three valuations extracted by the above procedures, a seven-day moving average scheme was also utilized as applied to the sentiment-valued time series. Thus, six distinct sentiment-valued time series were generated, the combinations of which, along with the *no-sentiment* and the univariate case scenario, led to the 22 different study cases. These, combined with the closing price data, constituted a single distinct experimental procedure for every algorithm. Below is a rough description of the three methods mentioned earlier:

- **TextBlob:** TextBlob is a Python-based framework for manipulating textual data. In this work, using the *sentiment* property from the above library, the polarity score—that is, a real number within the [−1,1] interval—was generated for every downloaded tweet. As has already been pointed out, a simple averaging scheme was then applied to the numerical output of the algorithm to produce a single sentiment value that represents the users’ attitude per day. The method, being a *rule-based* sentiment-analysis algorithm, works by calculating the value attributed to the corresponding sentiment score by simply applying a manually created set of rules. For example, counting the number of times a particular term appears in a given section adjusts the overall estimated sentiment score values in proportion to the way this term is evaluated;
- **Vader:** Vader is also a simple *rule-based* method for general sentiment analysis realization. The Vader Sentiment Analysis tool in practice works as follows: given a string—in this work, the textual elements of each tweet—*SentimentIntensityAnalyzer()* returns a dictionary, containing negative, neutral, and positive sentiment values, and a compound score produced by a normalization of the three latter. Again, maintaining only the “compound” value for each tweet, a normalized average of all such scores was generated for each day, resulting in a final time series that had those—ranging within the [−1,1] interval—daily sentiment scores as its values;
- **FinBERT:** FinBERT is a sentiment analysis pre-trained *natural-language-processing* (NLP) model that is produced by fine-tuning the BERT model over financial textual data. BERT, standing for *bidirectional encoder representations from transformers*, is an architecture for NLP problems based on the *transformers*. Multi-layer deep representations of linguistic data are trained under a bidirectional attention strategy from unlabeled data in a way that the contexts of each token constitute the content of its embedding. Moreover, targeting specific tasks, the model can be fine-tuned using just another layer. In essence, it is a pre-trained representational model, according to the principles of transfer learning. Here, using the implementation contained in [40], and especially the model trained on the *PhraseBank* presented in [41], the daily sentiment scores were extracted, and—according to the same pattern as before—a daily average was produced.

### 3.2. Algorithms

Now, regarding the algorithms used, it was already reported that 27 different methods were compared. From this, it is easy to conclude that it is practically impossible to present in detail such a number of algorithms in terms of their theoretical properties. Instead, a simple reference is provided while encouraging the reader to consult the corresponding citations for further information. Table 2 contains alphabetically all the algorithms used during the experimental process.

Experiments were run in the *Python* programming language using the *Keras* [67] open-source software library and *PyCaret* [68,69], an open-source, low-code machine-learning framework. It should also be noted that the problem of predicting the future values of the given time series was essentially addressed and consequently formalized as a regression problem. The forecasts were exported under one single-step and two multi-step prediction scenarios. Specifically, regarding multi-step forecasts, estimates were predicted for a seven-day window, on the one hand, and a fourteen-day window, on the other. All algorithms tested were utilized in a basic configuration with no optimization process taking place whatsoever.

### 3.3. Metrics

Moving on to the prediction performance estimates, given the comparative nature of the present work, the forthcoming description of the evaluation metrics to be presented is be a little more detailed. The following six metrics were used: MSE, RMSE, RMSLE, MAE, MAPE, and R${}^{2}$. The abbreviations are defined within the following subsections. Specifically, below is a presentation of these metrics, along with some insight regarding their interpretation. In what follows, the actual values of the observations are denoted by $y_{a_{i}}$ and the forecast values by $y_{p_{i}}$.

#### 3.3.1. MSE

The mean squared error (MSE) is simply the average of the squares of the differences between the actual values and the predicted values.

(1) $$\mathrm{MSE}=\frac{1}{n}\sum_{i=1}^{n}{\left(y_{p_{i}}-y_{a_{i}}\right)}^{2}$$

The square power ensures the absence of negative values while making small error information usable, i.e., minor deviations between the forecast and the actual values. It is evident, of course, that the greater the deviation of the predicted value from the actual one, the greater the penalty provided for under the MSE. A direct consequence of this is that the metric is greatly affected by the existence of outliers. Conversely, when the difference between the forecast and the actual value is less than one, the above interpretation works—in a sense—in reverse, resulting in an overestimation of the model’s predictive capacities. Because it is differentiable and can easily be optimized, the MSE constitutes a rather common forecast evaluation metric. It should be noted that the unit of measurement of the MSE is the square of the unit of measurement of the variable to predict.

#### 3.3.2. RMSE

The RMSE seems almost as an extension of the MSE. To compute it, one just calculates the root of the above.

(2) $$\mathrm{RMSE}=\sqrt{\frac{1}{n}\sum_{i=1}^{n}{\left(y_{p_{i}}-y_{a_{i}}\right)}^{2}}$$

That is, in our case, this is the quadratic mean (root mean square) of the differences between forecasts and actual, previously observed values. The formalization gives a representation of the average distance of the actual values from the predicted ones. The latter becomes easier to understand if one ignores the denominator in the formula: we observe that the formula is the same as that of the Euclidean distance, so dividing by the number *n* of the observations results in the RMSE being considered as some normalized distance. As with the MSE, the RMSE is affected by the existence of outliers. An essential role in the interpretability and, consequently, in the use of the RMSE is played by the fact that it is expressed in the same units with the target variable and not in its square, as in the MSE. It should also be noted that this metric is scale-dependent and can only be used to compare forecast errors of different models or model variations for a particular specific given variable.

#### 3.3.3. RMSLE

Below, in Equation (3), looking inside the square root, one notices that the RMSLE metric is a modified version of the MSE, a modification that is preferred in cases where the forecasts exhibit a significant deviation.

(3) $$\mathrm{RMSLE}=\sqrt{\frac{1}{n}\sum_{i=1}^{n}{\left(\log\left(y_{p_{i}}+1\right)-\log\left(y_{a_{i}}+1\right)\right)}^{2}}$$

As already mentioned, the MSE imposes a large “penalty” in cases where the forecast value deviates significantly from the actual value, a fact that the RMSLE compensates. As a result, this metric is resistant to the existence of both outliers, as well as noise. For this purpose, it utilizes the logarithms of the actual and the forecast value. The value of one is added to both the predicted and actual values in order to avoid cases where there is a logarithm of zero. It is straightforward that the RMSLE cannot be used when there exist negative values. Using the property: $\log\left(y_{p_{i}}+1\right)-\log\left(y_{a_{i}}+1\right)=log\left(\frac{y_{p_{i}}+1}{y_{a_{i}}+1}\right)$, it becomes clear that this metric actually works as the relative error between the actual value and the predicted value. It is worth noting that the RMSLE attributes more weight in cases where the predicted value is lower than the actual one than in cases where the forecast is higher than the observation. It is, therefore, particularly useful in certain types of forecasts (e.g., sales, where lower forecasts may lead to stock shortages if there is more than the projected demand).

#### 3.3.4. MAE

The MAE is probably the most straightforward metric to calculate. It is the arithmetic mean of the absolute errors (where the “*error*” is the difference between the predicted value and the actual value), assuming that all of them have the same weight.

(4) $$\mathrm{MAE}=\frac{1}{n}\sum_{i=1}^{n}\left|y_{p_{i}}-y_{a_{i}}\right|$$

The result is expressed (as in the RMSE) in the unit of measurement of the target variable. Regarding the existence of outliers, and given the absence of exponents in the formula, the MAE metric displays quite good behavior. Lastly, this metric—as the RMSE—depends on the scale of the observations. It can be used mainly to compare methods when predicting the same specific variable rather than different ones.

#### 3.3.5. MAPE

The MAPE stands for *mean absolute percentage error*. This metric is quite common for calculating the accuracy of forecasts, as it represents a relative and not an absolute error measure.

(5) $$\mathrm{MAPE}=\frac{1}{n}\sum_{i=1}^{n}\frac{\left|y_{p_{i}}-y_{a_{i}}\right|}{\left|y_{a_{i}}\right|}$$

A percentage represents accuracy: In Equation (5), we observe that the MAPE is calculated as the average of the absolute differences of the prediction from the actual value, divided by the observation. A multiplication by 100 can then transform the output value as a percentage. The MAPE cannot be calculated when the actual value is equal to zero. Moreover, it should be noted that if the forecast values are much higher than the actual ones, then the MAPE may exceed the 100% rate, while when both the prediction and the observation are low, it may not even approach 100%, leading to the erroneous conclusion that the predictive capacities of the model are limited, when in fact the error values may be low (Although, in theory, the MAPE is a percentage of 100, in practice, it can take values in $\left[0,\infty \right)$). The way it is calculated also tends to give more weight in cases where the predicted value is higher than the observation, thus leading to more significant errors. Therefore, there is a preference for using this metric in methods with low prediction values. Its main advantage is that it is not scale-dependent, so it can be used to evaluate comparisons of different time series, unlike the metrics presented above.

#### 3.3.6. R${}^{2}$

Lastly, the coefficient of determination $R^{2}$ is the ratio of the variance of the estimated values of the dependent variable to the fluctuation of the actual values of the dependent variable.

(6) $$R^{2}=1-\frac{SS_{RES}}{SS_{TOT}}=1-\frac{\sum_{i=1}^{n}{\left(y_{p_{i}}-y_{a_{i}}\right)}^{2}}{{\left(y_{p_{i}}-\bar{y}\right)}^{2}}$$

This metric is a measure of good fitting, as it attempts to quantify how well the regression model *fits* the data. Therefore, it is essentially not a measure of the reliability of the model. Typically, the values of $R^{2}$ range from 0–1. The value of zero corresponds to the case where the explanatory variables do not explain the variance of the dependent variable at all, while the value of one corresponds to the case where the explanatory variables fully explain the dependent variable. In other words, the closer the value of $R^{2}$ is to one, the better the model fits the observations (historical data), meaning the forecast values will be closer to the actual ones. However, there are cases where the output of $R^{2}$ goes beyond the above range and takes negative values. In this case (which is one allowed by its calculation formula), we conclude that our model has a worse performance (where “performance” means “data fitting”) than the simple horizontal line; in other words, the model does not follow the data trend. Concluding, values outside the above range—i.e., either greater than one or less than zero—either suggest the unsuitability of the model or indicate other errors in its implementation, such as the use of meaningless constraints.

## 4. Results and Discussion

Moving on to the results, as was already pointed out, the purpose of this work was twofold. The aim was to investigate two separate case studies through an extensive experimental procedure. Below are the results of the experiments categorized into these two separate cases. The first section deals with the utilization of textual data in light of sentiment analysis for the task of time series forecasting and the investigation of whether or not and when their use has a beneficial effect on improving predictions. The second involves comparing the performance of different forecast algorithms, aiming to fill the corresponding gap in the literature, where although there is serious research effort, it mainly concerns the comparison of a small number of methods. Table A1 presents the 22 sentiment score scenarios along with their respective abbreviations.

Apparently, the large number of experiments make any attempt to present numerical results in their raw form, that is, in the form of individual exported numerical predictions, impossible. It was therefore deemed necessary to use some performance measures that are well known and, in some ways, established in similar comparisons and capture the general behavior of each scenario. Moreover, it was already mentioned that the time series forecasting problem can be considered a regression one, and we see that in the present research—which presupposes a thorough study of the problem—six commonly accepted metrics were used. The choice of a number of various metrics was considered a necessary one, as each of them has advantages and disadvantages, presenting different aspects of the results that form a diverse set of guides for their evaluation.

Regarding aggregate comparisons, the first way of monitoring results to draw valid general conclusions was by the exploitation of the *Friedman ranking test* [70]. Thus, on the one hand, the H0 hypothesis—that is, whether all 22 different scenarios produce similar results—would have been tested, and on the other, it would have been made possible to classify the methods based on their efficiency. The Friedman statistical test is a non-parametric statistical test that checks whether the mean values of three or more *treatments*—in our case, the results of the twenty-two scenarios—differ significantly. Of the total six metrics used, five involved errors (MSE, RMSE, RMSLE, MAE, MAPE), which means that in order for one approach to be considered better than another, it must have a lower average. Therefore, the Friedman ranking error results follow an increasing order; the smaller the Friedman ranking score, the more efficient the method is. The opposite is the case only with $R^{2}$, where higher values indicate better performance.

After the Friedman test was performed, in case the null hypothesis was rejected—this rejection means that there is even one method that behaves differently—then the *Bonferroni–Dunn post hoc test* [71], also known as the Bonferroni inequality procedure, followed. This test generally reveals which pairs of treatments differ in their mean values, acting as follows: first the critical difference value is extracted, and then, for each pair of treatments, the absolute value of the difference in their rankings is calculated. If the latter is greater than or equal to the critical difference value, H0 is rejected, i.e., the corresponding treatments differ. The most efficient way to present the results of the Bonferroni inequality procedure is through *CD-diagrams*, where treatments whose performances do not differ are joined by horizontal dark lines. Below are tables with the results of the Friedman tests, boxplots with the error distributions, as well as CD-diagrams, which, due to the limited space available, show the relations between the top-10 best approaches according to the Friedman rankings.

### 4.1. Case Study: Sentiment Scores’ Comparison

Let us initially give a summary of the case. First, the aim was to answer whether and under what conditions the use of sentiment analysis in data derived from social media has a positive effect on the prediction of future prices of financial time series. Here, the combinations—seen in Table A1—of scores from three different sentiment analysis methods together with their seven-day rolling means and the univariate case created a total of twenty-two cases to compare. Table A2, Table A3 and Table A4 present the final Friedman rankings in terms of their corresponding single-day, seven-day and fourteen-day forecasts.

#### 4.1.1. Single-Day Prediction

First, regarding the forecast for the next day only, Table A2 shows the general superiority of the univariate case over the use of sentiment analysis. As for the boxplots and CD-diagrams, the top-ten combinations of sentiment time series for each metric presented are ranked with the same performance dominance of the univariate scenario (note that in boxplots, the top-down layout is sorted by median).

**Figure 3:**
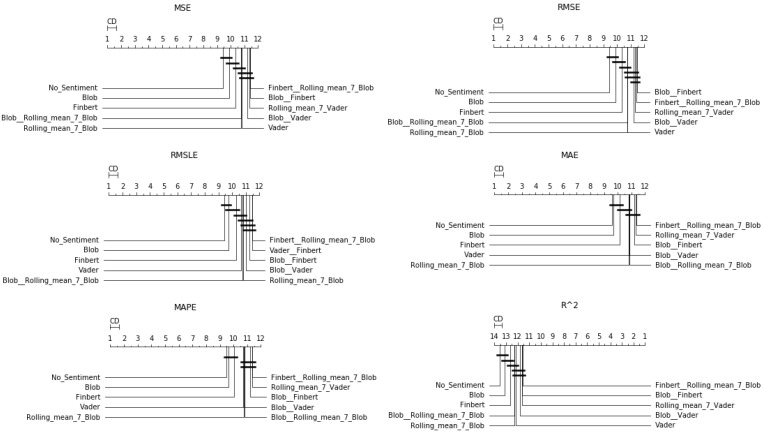
Sentiment setups’ CD-diagrams: *single-day prediction*.

**Figure 4:**
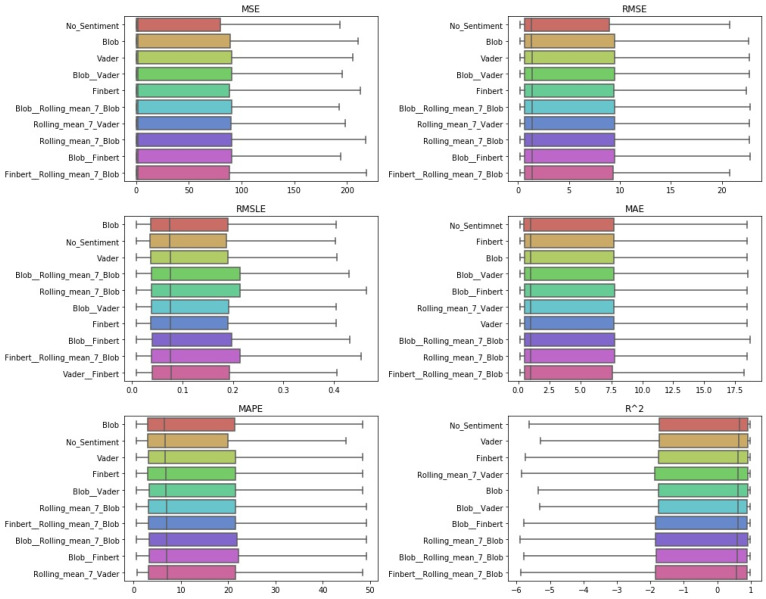
Sentiment setups’ boxplots: *single-day prediction*.

One can also observe the statistical dependencies that emerged from the examination of each pair of cases. These dependencies can be further analyzed by comparing Table A2 with the representations in Figure 3. For example, it was observed that the statistical dependence of the univariate case with that of the additional use of TextBlob shown in Figure 3 followed the ranking of the two versions extracted from the results in the Friedman tables. Figure 4 shows the performance distributions for each sentiment setup, i.e., all the values that resulted from applying a given setting to each dataset for each algorithm. Here, the apparent similarity of the performances of the methods is, on the one hand, a matter of the scale of the representation, while on the other, it reflects a possible uniformity. From all three different representations of the results, there was a predominance of the univariate version followed by the use of TextBlob and FinBERT.

#### 4.1.2. One-Week Prediction

**Figure 5:**
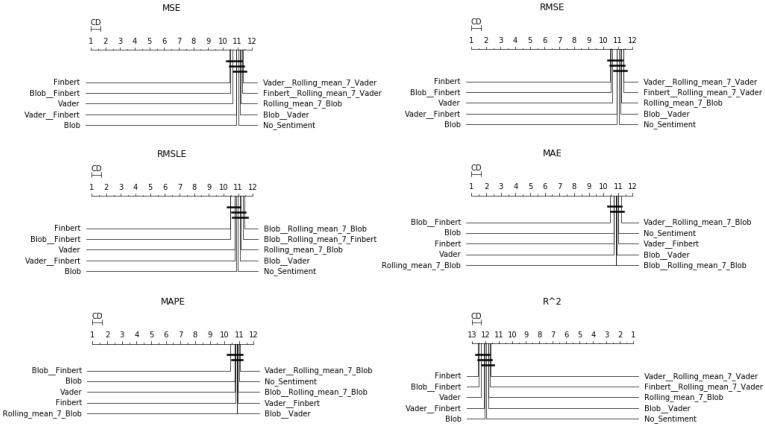
Sentiment setups’ CD-diagrams: *one-week prediction*.

**Figure 6:**
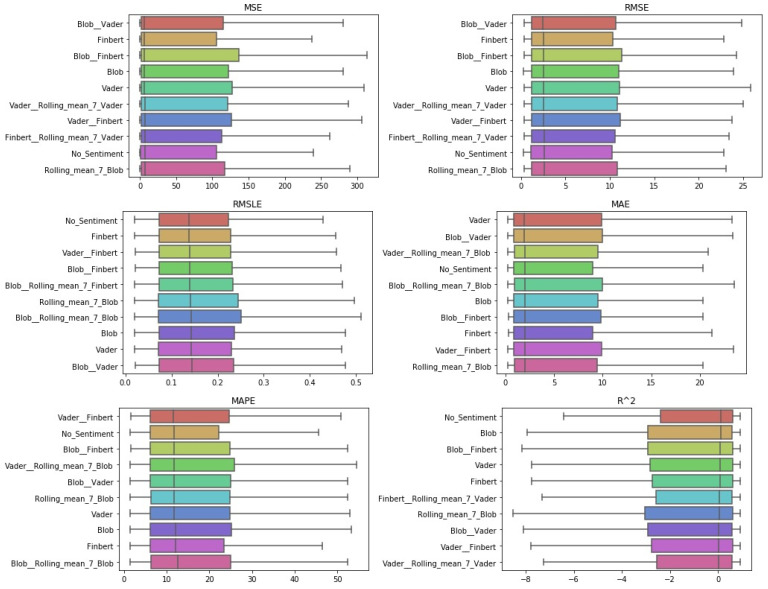
Sentiment setups’ boxplots: *one-week prediction*.

However, in the case of weekly forecasts, one can observe, from Table A3 and Figure 5 and Figure 6, that things do not remain the same. There was a noticeable decline in the performance ranking of the univariate setup, with the simultaneous improvement of configurations that utilize sentiment scores.

In particular, in four of the measurements used, FinBERT seemed to be superior, while in the other two, the combination of FinBERT with TextBlob lied in the first place of the ranking. Apart from that, Vader, Blob, and the combination of Vader and FinBERT seemed to perform almost equal to the above, as the differences in their corresponding rankings were minimal. In addition, regarding the use of rolling means, there seemed to be no particular improvement under the current framework except—in rare cases—when applied in combination with the use of a raw sentiment score. The only one of the representations of the results where the univariate configuration is presented in high positions is via boxplots, where the sorting of the layout is only based on the median of the values. In terms of Friedman scores, at best, it ranked sixth.

#### 4.1.3. Two-Week Prediction

**Figure 7:**
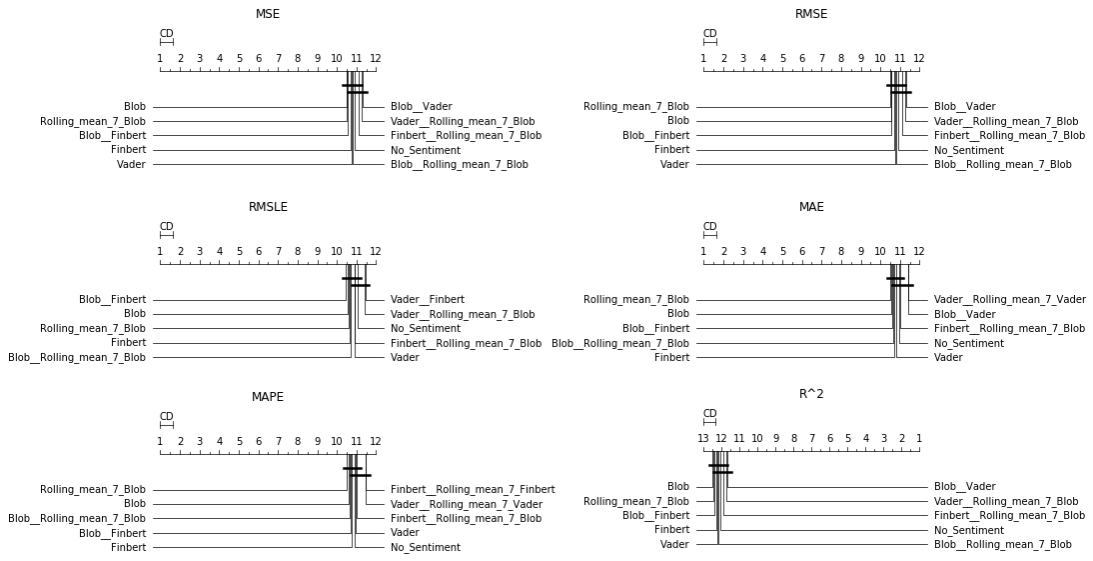
Sentiment setups’ CD-diagrams: *two-week prediction*.

**Figure 8:**
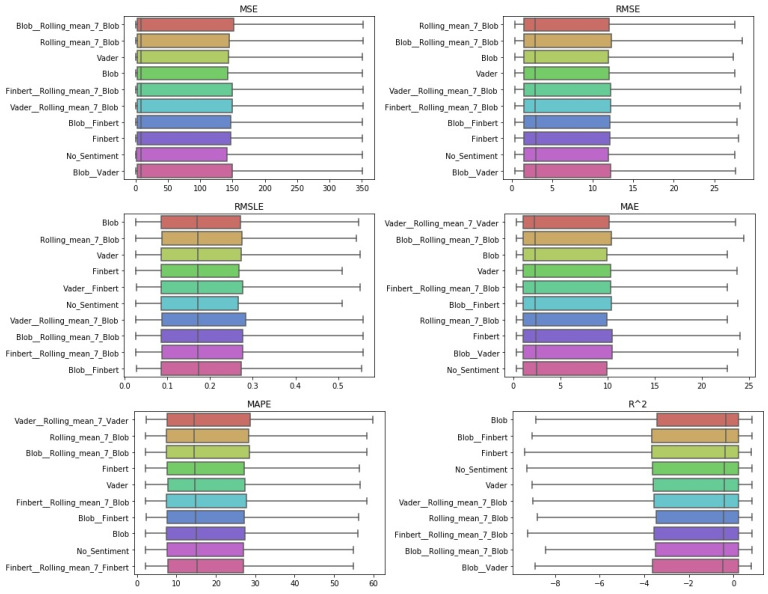
Sentiment setups’ boxplots: *two-week prediction*.

Results from the fourteen-day forecasts exhibited similar behavior as in the seven-day prediction case, except for the performance of the averaging schemes, some of which tended to move up to higher positions. Indeed, here, again, Friedman’s ranking in all evaluations seemed to suggest that the use of information extracted from social networks is beneficial under the current forecasting framework. In addition, there was an apparent improvement in schemes exploiting rolling means. This becomes easily noticeable in both Figure 7 and Figure 8, showing the CD-diagrams and boxplots, respectively, and in Table A4. One can observe the configuration of TextBlob that incorporates the weekly rolling mean to be in the first place of the Friedman ranking in terms of three valuations, that is in terms of the RMSE, MAE, and MAPE metrics. Thus, apart from the conclusions that can be drawn from the study of the representations of the results and that constitute evaluations similar in form to those of the above cases, something new seemed to emerge here: there was a gradual increase in the performance of the combinations that use weighted information. Moreover, this increase in performance seemed to be related to the long forecast period.

### 4.2. Case Study: Methods’ Comparison

We can now turn to the presentation of the results of the comparison of the algorithms. The reader is first asked to refer to Table 2, containing the methods with their respective abbreviations, as well as to Table A5, Table A6 and Table A7, containing the Friedman rankings. The Friedman rankings here are structured as a generalization derived from the performance of each algorithm in terms of each dataset and under each of the 22 input schemes.

#### 4.2.1. One-Day Prediction

**Figure 9:**
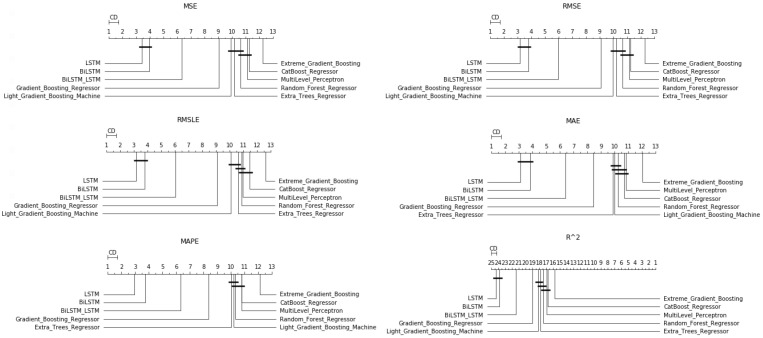
Algorithms’ CD-diagrams: *single-day prediction*.

**Figure 10:**
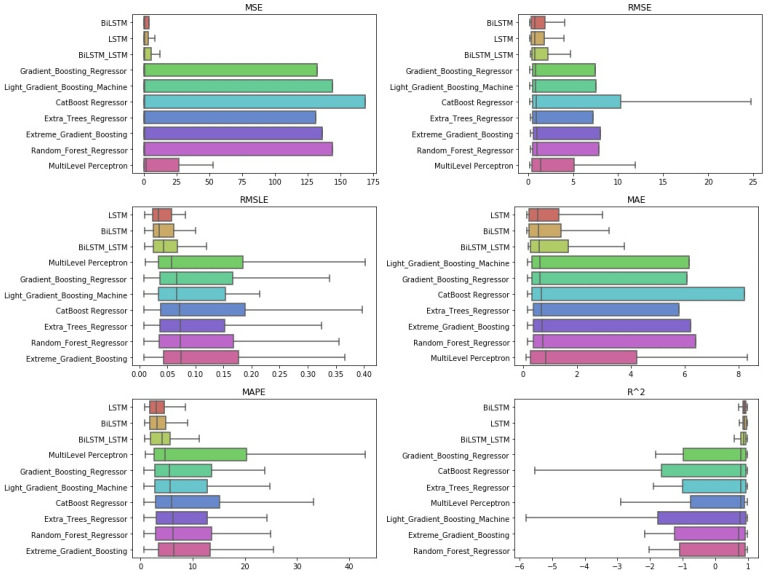
Algorithms’ boxplots: *single-day prediction*.

Starting with the simple one-day prediction, from the results presented in Table A5 and in Figure 9 and Figure 10, one can easily conclude an almost universal predominance of LSTM methods.

Regarding the three best-performing methods, the CD-diagrams show a statistical dependence between the LSTM and Bi-LSTM methods, while the scheme incorporating both the above algorithmic processes in a stacked configuration is presented as statistically independent of all. This supposed independence, and according to what has been reported about how these diagrams are derived, can easily be identified in the differences in the results of the Friedman table, where the deviations between the methods are significant.

The latter is eminent in the boxplots as well. Both the dispersion and the values of the evaluations of the top-three methods stand out clearly from those of all the other techniques.

#### 4.2.2. One-Week Prediction

**Figure 11:**
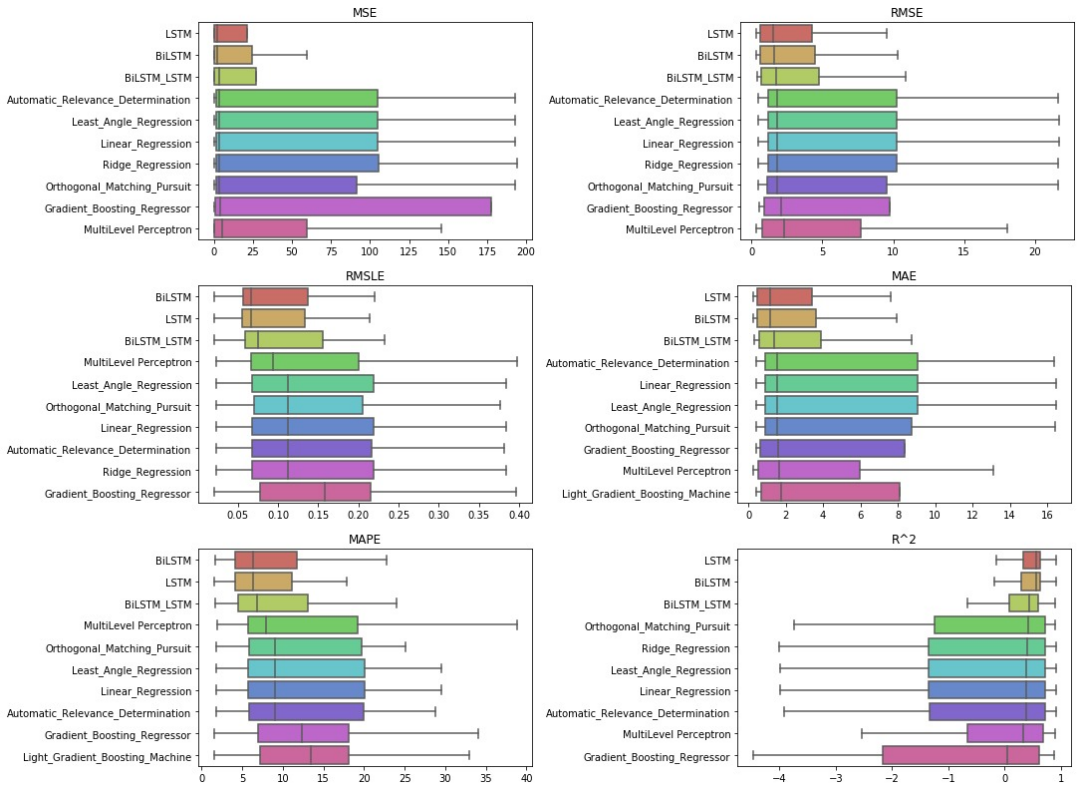
Algorithms’ boxplots: *one-week prediction*.

**Figure 12:**
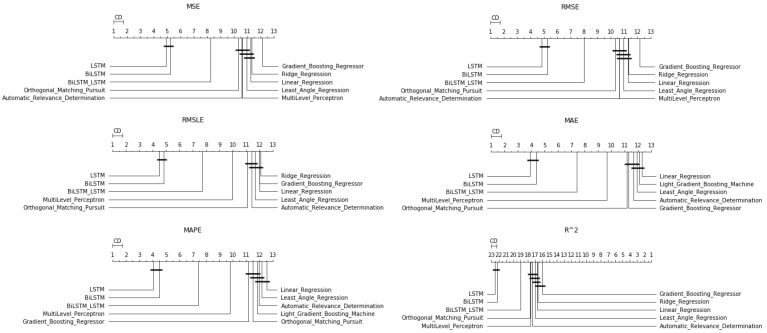
Algorithms’ CD-diagrams: *one-week prediction*.

It can be observed that the same interpretation applies in the case of weekly forecasts. Again, in all metrics, the top-three best-performing methods were the three LSTM variants (Figure 11). Table A6 depicts both the latter and the distinctions presented on the CD-diagrams of Figure 12. Essentially, however, a simple comparison of the representations of the results showed that in all cases, the predominant methods were by far the LSTM and Bi-LSTM procedures.

In the boxplots, despite the fact that the LSTM variants appear as if they tend to form a group of similarly performing methods, the Friedman scores point to the independence—in terms of the evaluation of numerical outputs—of only the top-two aforementioned methods from all the others. Thus, based on these results, it is relatively easy to suggest a clear choice of strategy in terms of methods.

#### 4.2.3. Two-Week Prediction

**Figure 13:**
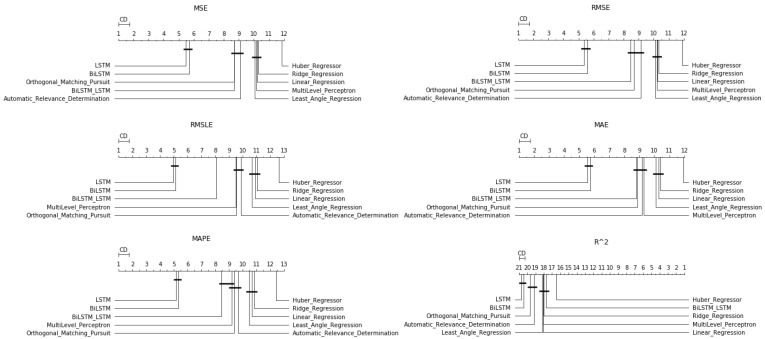
Algorithms’ CD-diagrams: *two-week prediction*.

**Figure 14:**
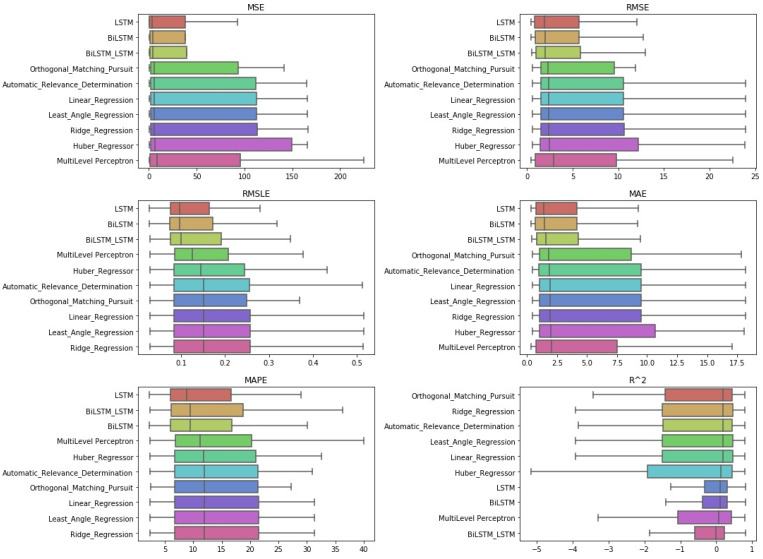
Algorithms’ boxplots: *two-week prediction*.

Finally, regarding the case of the 14-day forecasts, the general remarks given in the previous section can be extended here as well. The results can be found below, in Figure 13 and Figure 14, as well as in Table A7.

An additional final remark, however, should be the following: in the boxplots, in the results of the $R^{2}$, there seems to be a difference in the median ranking. This ranking, however, was not found in the case of the Friedman scores.

### 4.3. Discussion

Having presented the results, below are some general remarks. Here, the following discussion is structured according to the bilateral distinction of the case studies presented and contains summarizing comments regarding elements that preceded:

- **Sentiment setups:** The main point that emerged from the above results has to do with the fact that the use of sentiment analysis seemed to improve the models when used for long-term predictions. Thus, while the use of the univariate configuration is seen as more efficient in one-step predictions, when the predictions applied to the seven-day and fourteen-day cases, the use of sentiment scores under a multivariate topology seemed to improve the forecasts overall. Specifically, in the weekly forecasts, all three single-sentiment-score setups outperformed the use of the univariate configuration, with FinBERT performing best in terms of the MSE, RMSE, RMSLE, and $R^{2}$, while the combination of Blob and FinBERT outperformed the rest in the MAE and MAPE. When the prediction shift doubles to 14 days, one notices that Blob and Rolling Mean 7 Blob dominated the other sentiment configurations, followed by the combination of Blob and FinBERT, as well as FinBERT. Vader appeared to rank lower in all metrics and was, therefore, weaker than in the previous two cases.
  However, two general questions need to and can be answered by looking at the results. These are not about choosing an algorithm, as one can assume that in a working scenario where reliable predictions would be needed, one would have a number of methods at one’s disposal. Thus, this is a query about a reliable methodology. Therefore, first of all, one should evaluate whether the use of sentiment scores helps and, if so, in which cases. Second, an answer must be provided as to what form the sentiment score time series should have depending on the forecasting case. Regarding the first question, the answer seems to be clear: multivariate configurations improve forecasts in non-trivial forecast cases. As for the second one, it seems that, in cases of long-term forecasts, an argument in favor of the use of rolling mean can be substantiated. Concluding, it should be noted that when the forecast window grows, then even seemingly small improvements, such as those seen through the use of sentiment analysis, can be of particular importance;
- **Algorithms:** As for the algorithms, the comparisons seemed to provide direct and clear interpretations. From the results here, it is also possible to safely substantiate—at least—a central conclusion. It is apparent that in all scenarios, the configurations exploiting neural networks—that is, LSTM variations—were superior in terms of performance to the classical regression algorithms. Among them, LSTM outperformed the BiLSTM architecture in every single case, while the stacked combination of the two followed. In addition, the aforementioned superiority of the two dominant methods was clear, with their performance forming a threshold, below which—and at a considerable distance—all the other methods examined were placed. Therefore, concluding, if one considers that the neural network architectures used did not contain sophisticated configurations—in terms of, for example, depth—then, on the basis that any additional computational costs become negligible, the use of LSTMs constitutes the clear choice.

## 5. Conclusions

In this work, a study of the exploitation of sentiment scores in various multivariate time-series-forecasting schemes regarding financial data was conducted. The overall structure and results of an extensive experimental procedure were presented, in which 22 different input configurations were tested, utilizing information extracted from social networks, in a total of 16 different datasets, using 27 different algorithms. The survey consisted of two case studies, the first of which was to investigate the performance of various multivariate time series forecasting schemes utilizing sentiment analysis and the second to compare the performance of a large number of machine-learning algorithms using the aforementioned multivariate input setups.

From the results, and in relation to the first case study, that is, after the use of sentiment analysis configurations, a conditional performance improvement can be safely deduced in cases where the methods were applied to predict long-term time frames. Of all the sentiment score combinations tested, the TextBlob and FinBERT variations generally appeared to perform best. In addition, there was a gradual improvement in the performance of combinations containing rolling averages as the forecast window grew. This may imply that a broader study of the use of different versions of the same time series in a range of different multivariate configurations may reveal methodological strategies as to how to exploit input data manipulations to increase accuracy.

Regarding the second case study, the results indicated a clear predominance of LSTM variations. In particular, this superiority became even clearer in terms of its generalization when the basic configurations of the architectures used in the neural networks under consideration were taken into account, which means that any computational cost cannot be a counterweight to the dominance of the LSTM methods.

## Figures and Tables

**Table 1:** Stock datasets.

No	Dataset	Stocks
1	AAL	American Airlines Group
2	AMD	Advanced Micro Devices
3	AUY	Yamana Gold Inc.
4	BABA	Alibaba Group
5	BAC	Bank of America Corp.
6	ET	Energy Transfer L.P.
7	FCEL	FuelCell Energy Inc.
8	GE	General Electric
9	GM	General Motors
10	INTC	Intel Corporation
11	MRO	Marathon Oil Corporation
12	MSFT	Microsoft
13	OXY	Occidental Petroleum Corporation
14	RYCEY	Rolls-Royce Holdings
15	SQ	Square
16	VZ	Verizon Communications

**Table 2:** Algorithms.

No.	Abbreviation	Algorithm
1	ABR	AdaBoost Regressor [42]
2	ARD	Automatic Relevance Determination [43]
3	BiLSTM (LSTM_2)	Bidirectional LSTM [44]
4	BiLSTM-LSTM (LSTM_3)	Bidirectional LSTM and LSTM Stacked [44,45]
5	CBR	CatBoost Regressor [46]
6	DTR	Decision Tree Regressor [47]
7	ELN	Elastic Net [48]
8	ET	Extra Trees Regressor [49]
9	XGBoost	Extreme Gradient Boosting [50]
10	GB	Gradient Boosting Regressor [51]
11	HBR	Huber Regressor [52]
12	KNR	K-Neighbors Regressor [53]
13	KER	Kernel Ridge [54]
14	LSTM	LSTM [45]
15	LA-LAS	Lasso Least Angle Regression [55]
16	LAS	Lasso Regression [56]
17	LA	Least Angle Regression [55]
18	LGBM	Light Gradient Boosting Machine [57]
19	LNR	Linear Regression [58]
20	MLP	Multilevel Perceptron [59]
21	OMP	Orthogonal Matching Pursuit [60]
22	PAR	Passive Aggressive Regressor [61]
23	RF	Random Forest Regressor [62]
24	RSC	Random Sample Consensus [63]
25	RDG	Ridge Regression [64]
26	SVR	Support Vector Regression [65]
27	THS	Theil–Sen Regressor [66]

## Data Availability

*URL* of the full Friedman Ranking results: https://bit.ly/2XlBNvL (accessed on 1 November 2021).

## Appendix A. Friedman Rankings

Some of the results in the tables below are slightly truncated due to space limitations. The full results of the Friedman rankings can be found at the following URL: https://bit.ly/2XlBNvL (accessed on 1 November 2021).

### Appendix A.1. Sentiments

**Table A1:** Sentiment score setups.

No.	Abbreviation	Sentiment Score Setup
1	NS	No Sentiment
2	B	TextBlob
3	V	Vader
4	F	FinBERT
5	B7	Rolling Mean 7 TextBlob
6	V7	Rolling Mean 7 Vader
7	F7	Rolling Mean 7 FinBERT
8	BV	TextBlob and Vader
9	BF	TextBlob and FinBERT
10	BB7	TextBlob and Rolling Mean 7 TextBlob
11	BV7	TextBlob and Rolling Mean 7 Vader
12	BF7	TextBlob and Rolling Mean 7 FinBERT
13	VF	Vader and FinBERT
14	VB7	Vader and Rolling Mean 7 TextBlob
15	VV7	Vader and Rolling Mean 7 Vader
16	VF7	Vader and Rolling Mean 7 FinBERT
17	FB7	FinBERT and Rolling Mean 7 TextBlob
18	FV7	FinBERT and Rolling Mean 7 Vader
19	FF7	FinBERT and Rolling Mean 7 FinBERT
20	B7V7	Rolling Mean 7 TextBlob and Rolling Mean 7 Vader
21	B7F7	Rolling Mean 7 TextBlob and Rolling Mean 7 FinBERT
22	V7F7	Rolling Mean 7 Vader and Rolling Mean 7 FinBERT

**Table A2:** Sentiment scenarios’ Friedman rankings (shift = 1).

	MSE		RMSE		RMSLE	
	**Setup**	**F-Rank**	**Setup**	**F-Rank**	**Setup**	**F-Rank**
1	NS	9.445601852	NS	9.440972222	NS	9.420138889
2	B	10.35763889	B	10.35763889	B	9.710648148
3	F	10.3576389	F	10.3576389	F	10.30092593
4	BB7	10.73263889	BB7	10.73032407	V	10.64351852
5	B7	10.74305556	B7	10.74537037	BB7	10.77546296
6	V	10.81018519	V	10.77546296	B7	10.81365741
7	BV	11.22569444	BV	11.19560185	BV	10.99768519
8	V7	11.35416667	V7	11.33564815	BF	11.28356481
9	BF	11.40740741	FB7	11.42013889	VF	11.44212963
10	FB7	11.42361111	BF	11.44444444	FB7	11.48842593
11	VB7	11.43634259	VB7	11.4525463	V7	11.50694444
12	VF	11.48148148	VF	11.50231481	VB7	11.57060185
13	VV7	11.66087963	VV7	11.66550926	F7	11.66782407
14	F7	11.76967593	F7	11.77199074	FF7	11.78125
15	FF7	11.84490741	FF7	11.83449074	VV7	11.92013889
16	BV7	12.01967593	BV7	11.97800926	BV7	12.18402778
17	BF7	12.15740741	BF7	12.2037037	BF7	12.18865741
18	VF7	12.28009259	VF7	12.2662037	VF7	12.21527778
19	FV7	12.46759259	FV7	12.51157407	B7V7	12.61689815
20	B7F7	12.78587963	B7F7	12.76967593	FV7	12.7337963
21	V7F7	12.85532407	B7V7	12.85763889	B7F7	12.78009259
22	B7V7	12.86689815	V7F7	12.86226852	V7F7	12.95833333
	**MAE**		**MAPE**		$R^{2}$	
	**Setup**	**F-Rank**	**Setup**	**F-Rank**	**Setup**	**F-Rank**
1	NS	9.591435185	NS	9.503472222	NS	13.55208333
2	B	9.688657407	B	9.616898148	B	13.12615741
3	F	10.16782407	F	10.05671296	F	12.6400463
4	V	10.79050926	V	10.72337963	BB7	12.26736111
5	B7	10.82175926	B7	10.73842593	B7	12.25810185
6	BB7	10.85532407	BB7	10.78356481	V	12.19097222
7	BV	10.8599537	BV	10.79398148	BV	11.77430556
8	BF	11.21759259	BF	11.19907407	V7	11.64930556
9	V7	11.32986111	V7	11.36574074	BF	11.59259259
10	FB7	11.38773148	FB7	11.37615741	FB7	11.57638889
11	VF	11.48611111	VF	11.5	VB7	11.56365741
12	VB7	11.6400463	VB7	11.66898148	VF	11.51851852
13	F7	11.69791667	FF7	11.72569444	VV7	11.33680556
14	FF7	11.69791667	F7	11.80324074	F7	11.22916667
15	VV7	11.87847222	VV7	11.92476852	FF7	11.15625
16	BV7	12.11689815	BV7	12.1875	BV7	10.98032407
17	BF7	12.19212963	BF7	12.28240741	BF7	10.84259259
18	VF7	12.34027778	VF7	12.28240741	VF7	10.72106481
19	FV7	12.35648148	FV7	12.42013889	FV7	10.53240741
20	B7F7	12.72337963	B7F7	12.64699074	B7F7	10.21412037
21	V7F7	13.01041667	V7F7	13.09953704	V7F7	10.14467593
22	B7V7	13.14930556	B7V7	13.17592593	B7V7	10.13310185

**Table A3:** Sentiment scenarios’ Friedman rankings (shift = 7).

	MSE		RMSE		RMSLE	
	**Setup**	**F-Rank**	**Setup**	**F-Rank**	**Setup**	**F-Rank**
1	F	10.45486111	F	10.46875	F	10.44444444
2	BF	10.5162037	BF	10.53009259	BF	10.46412037
3	V	10.64930556	V	10.62847222	V	10.75231481
4	VF	10.90509259	VF	10.91203704	VF	10.78356481
5	B	10.9224537	B	10.91782407	B	10.84953704
6	NS	11.06828704	NS	11.09375	NS	10.96064815
7	BV	11.19907407	BV	11.18518519	BV	11.14583333
8	B7	11.23263889	B7	11.23958333	B7	11.16087963
9	FV7	11.34143519	FV7	11.36689815	BF7	11.30902778
10	VV7	11.3900463	VV7	11.40162037	BB7	11.42476852
11	FF7	11.52199074	FF7	11.52083333	FF7	11.44444444
12	BF7	11.54398148	BF7	11.52314815	VB7	11.47685185
13	BB7	11.5625	BB7	11.54398148	FB7	11.52430556
14	FB7	11.6087963	FB7	11.62384259	VV7	11.67708333
15	BV7	11.71064815	BV7	11.69907407	VF7	11.72222222
16	VB7	11.73958333	VB7	11.73958333	FV7	11.74421296
17	V7	11.76967593	V7	11.7650463	BV7	11.89814815
18	VF7	11.87847222	VF7	11.85300926	F7	12.16782407
19	F7	12.1412037	F7	12.14583333	V7	12.23842593
20	B7V7	12.54861111	B7V7	12.55555556	B7V7	12.52546296
21	V7F7	12.64236111	V7F7	12.62847222	B7F7	12.56944444
22	B7F7	12.65277778	B7F7	12.65740741	V7F7	12.71643519
	**MAE**		**MAPE**		$R^{2}$	
	**Setup**	**F-Rank**	**Setup**	**F-Rank**	**Setup**	**F-Rank**
1	BF	10.45949074	BF	10.38078704	F	12.54513889
2	B	10.67939815	B	10.67824074	BF	12.4849537
3	F	10.68634259	V	10.70023148	V	12.35069444
4	V	10.71064815	F	10.73958333	VF	12.09490741
5	B7	10.85532407	B7	10.85300926	B	12.0775463
6	BB7	10.87847222	BV	10.86921296	NS	11.93171296
7	BV	10.88310185	VF	10.90162037	BV	11.80092593
8	VF	10.9849537	BB7	10.92476852	B7	11.76736111
9	NS	11.02893519	NS	11.00925926	FV7	11.65972222
10	VB7	11.1875	VB7	11.03472222	VV7	11.6099537
11	FB7	11.42013889	FB7	11.42708333	FF7	11.47800926
12	BF7	11.62037037	BF7	11.52314815	BF7	11.4537037
13	VV7	11.74189815	VV7	11.7650463	BB7	11.43865741
14	FF7	11.77314815	FF7	11.78009259	FB7	11.3912037
15	FV7	11.85300926	VF7	11.90046296	BV7	11.28587963
16	VF7	11.90972222	FV7	12.0474537	VB7	11.26041667
17	BV7	11.91319444	BV7	12.08101852	V7	11.23032407
18	V7	12.17592593	V7	12.13078704	VF7	11.12152778
19	F7	12.26388889	F7	12.22685185	F7	10.8587963
20	B7F7	12.5	B7F7	12.53935185	B7V7	10.4525463
21	B7V7	12.63310185	B7V7	12.54166667	V7F7	10.3587963
22	V7F7	12.84143519	V7F7	12.94560185	B7F7	10.34722222

**Table A4:** Sentiment scenarios’ Friedman rankings (shift = 14).

	MSE		RMSE		RMSLE	
	**Setup**	**F-Rank**	**Setup**	**F-Rank**	**Setup**	**F-Rank**
1	B	10.48726852	B7	10.50810185	BF	10.46527778
2	B7	10.50462963	B	10.52777778	B	10.53125
3	BF	10.56597222	BF	10.55208333	B7	10.60300926
4	F	10.70949074	F	10.71990741	F	10.66550926
5	V	10.75925926	V	10.74884259	BB7	10.70717593
6	BB7	10.7974537	BB7	10.80208333	V	10.92476852
7	NS	10.92592593	NS	10.89583333	FB7	10.92939815
8	FB7	11.10185185	FB7	11.09027778	NS	11.04976852
9	VB7	11.25694444	VB7	11.28240741	VB7	11.41319444
10	BV	11.3275463	BV	11.3275463	VF	11.45601852
11	VF	11.37615741	VF	11.37152778	FF7	11.46296296
12	VV7	11.52777778	VV7	11.52083333	BV	11.46990741
13	V7	11.70023148	V7	11.71064815	VV7	11.75578704
14	FF7	11.75925926	FF7	11.75231481	BF7	11.78240741
15	BV7	11.84722222	BV7	11.84143519	BV7	11.87268519
16	BF7	12.00115741	BF7	11.99652778	F7	11.92708333
17	B7F7	12.01851852	B7F7	12.02083333	V7	11.94560185
18	B7V7	12.05092593	FV7	12.05671296	FV7	12.12847222
19	FV7	12.0625	B7V7	12.06597222	B7F7	12.15509259
20	F7	12.30555556	F7	12.30671296	VF7	12.21180556
21	VF7	12.47106481	VF7	12.45486111	B7V7	12.27546296
22	V7F7	13.44328704	V7F7	13.44675926	V7F7	13.26736111
	**MAE**		**MAPE**		$R^{2}$	
	**Setup**	**F-Rank**	**Setup**	**F-Rank**	**Setup**	**F-Rank**
1	B7	10.51157407	B7	10.50694444	B	12.50925926
2	B	10.53125	B	10.60532407	B7	12.49652778
3	BF	10.59490741	BB7	10.64351852	BF	12.43171296
4	BB7	10.62962963	BF	10.70601852	F	12.29166667
5	F	10.69328704	F	10.74189815	V	12.24074074
6	V	10.79513889	NS	10.88425926	BB7	12.20717593
7	NS	10.94907407	V	10.93634259	NS	12.07407407
8	FB7	10.99421296	FB7	11.03356481	FB7	11.89583333
9	BV	11.41087963	VV7	11.44212963	VB7	11.74305556
10	VV7	11.42824074	FF7	11.47337963	BV	11.6724537
11	V7	11.43055556	VB7	11.49537037	VF	11.62384259
12	VB7	11.44675926	V7	11.50925926	VV7	11.47222222
13	VF	11.59375	VF	11.66319444	V7	11.29976852
14	BV7	11.6400463	BV	11.68634259	FF7	11.23842593
15	FF7	11.67476852	FV7	11.80208333	BV7	11.15277778
16	FV7	11.80902778	BF7	11.81944444	BF7	11.00347222
17	BF7	12.00231481	BV7	11.86458333	B7F7	10.97916667
18	B7F7	12.17013889	B7F7	12.10763889	B7V7	10.94907407
19	F7	12.39351852	F7	12.14583333	FV7	10.9375
20	B7V7	12.46643519	VF7	12.36689815	F7	10.69560185
21	VF7	12.52662037	B7V7	12.53587963	VF7	10.52893519
22	V7F7	13.30787037	V7F7	13.03009259	V7F7	9.556712963

### Appendix A.2. Algorithms

**Table A5:** Methods’ Friedman rankings (shift = 1).

	MSE		RMSE		RMSLE	
	**Method**	**F-Rank**	**Method**	**F-Rank**	**Method**	**F-Rank**
1	LSTM	3.409090909	LSTM	3.184659091	LSTM	3.178977273
2	LSTM_2	3.954545455	LSTM_2	3.786931818	LSTM_2	3.801136364
3	LSTM_3	6.34375	LSTM_3	5.977272727	LSTM_3	6.065340909
4	GB	9.048295455	GB	9.076704545	GB	9.105113636
5	LGBM	9.923295455	LGBM	9.96875	LGBM	10.10511364
6	ET	10.17045455	ET	10.22443182	ET	10.625
7	RF	10.64488636	RF	10.6875	RF	10.87215909
8	MLP	11.13636364	MLP	11.17897727	MLP	10.95738636
9	CBR	11.23863636	CBR	11.25852273	CBR	11.47443182
10	XGBoost	12.26420455	XGBoost	12.30397727	XGBoost	12.63352273
11	ARD	13.11647727	ARD	13.16761364	ARD	13.5625
12	OMP	13.21164773	OMP	13.26846591	OMP	13.70596591
13	LA	13.66619318	LA	13.71164773	LA	14.18323864
14	RDG	13.77272727	RDG	13.81818182	RDG	14.21875
15	LNR	13.78267045	LNR	13.828125	LNR	14.29829545
16	ABR	14.68465909	ABR	14.71022727	ABR	15.11079545
17	DTR	15.34090909	DTR	15.36079545	DTR	15.64772727
18	KNR	16.19034091	KNR	16.21022727	KNR	15.99431818
19	RSC	16.31676136	RSC	16.35085227	RSC	16.50568182
20	HBR	16.91477273	HBR	16.96022727	HBR	17.30397727
21	SVR	17.85511364	SVR	17.86079545	THS	17.51704545
22	THS	18.11647727	LAS	18.13068182	SVR	17.63068182
	**MAE**		**MAPE**		$R^{2}$	
	**Method**	**F-Rank**	**Method**	**F-Rank**	**Method**	**F-Rank**
1	LSTM	3.113636364	LSTM	2.960227273	LSTM	24.34375
2	LSTM_2	3.835227273	LSTM_2	3.747159091	LSTM_2	23.79545455
3	LSTM_3	6.423295455	LSTM_3	6.346590909	LSTM_3	21.36931818
4	GB	8.448863636	GB	8.360795455	GB	19.00568182
5	ET	9.889204545	ET	10.01988636	LGBM	18.15340909
6	LGBM	9.997159091	LGBM	10.21875	ET	17.84943182
7	RF	10.24431818	RF	10.34943182	RF	17.38636364
8	CBR	10.69318182	MLP	10.78409091	MLP	16.88636364
9	MLP	10.81818182	CBR	10.80681818	CBR	16.76988636
10	XGBoost	12.02840909	XGBoost	12.11079545	XGBoost	15.75568182
11	ARD	13.83522727	ARD	13.75568182	ARD	14.94602273
12	OMP	13.96164773	OMP	13.87642045	OMP	14.84517045
13	LA	14.20880682	LA	14.140625	LA	14.40198864
14	RDG	14.3125	RDG	14.28125	RDG	14.29545455
15	LNR	14.34943182	LNR	14.29119318	LNR	14.28551136
16	ABR	14.67613636	ABR	14.69318182	ABR	13.33806818
17	DTR	14.94034091	DTR	15.05113636	DTR	12.66477273
18	KNR	15.53693182	KNR	15.36931818	KNR	11.85227273
19	SVR	15.73579545	SVR	15.84375	RSC	11.73153409
20	RSC	16.81534091	RSC	16.77982955	HBR	11.13352273
21	HBR	17.45170455	HBR	17.44034091	SVR	10.17045455
22	THS	18.65340909	LAS	18.62215909	THS	9.909090909

**Table A6:** Methods’ Friedman rankings (shift = 7).

	MSE		RMSE		RMSLE	
	**Method**	**F-Rank**	**Method**	**F-Rank**	**Method**	**F-Rank**
1	LSTM	4.940340909	LSTM	4.852272727	LSTM	4.491477273
2	LSTM_2	5.272727273	LSTM_2	5.235795455	LSTM_2	4.823863636
3	LSTM_3	8.241477273	LSTM_3	8.005681818	LSTM_3	7.696022727
4	OMP	10.31960227	OMP	10.33664773	MLP	9.9375
5	ARD	10.60511364	ARD	10.61931818	OMP	11.07528409
6	MLP	10.61931818	MLP	10.64488636	ARD	11.38068182
7	LA	10.96732955	LA	10.98153409	LA	11.63778409
8	LNR	11.26988636	LNR	11.28409091	LNR	11.98295455
9	RDG	11.34375	RDG	11.35795455	GB	11.98863636
10	GB	12.14772727	GB	12.17329545	RDG	12.05397727
11	LGBM	12.72443182	LGBM	12.75	LGBM	12.91193182
12	ET	13.65909091	ET	13.68181818	ABR	13.69034091
13	HBR	13.67613636	HBR	13.69318182	ET	13.88920455
14	CBR	13.90909091	CBR	13.90909091	CBR	13.89488636
15	ABR	14.25284091	ABR	14.27272727	RF	14.48295455
16	RF	14.38920455	RF	14.40056818	THS	14.59232955
17	THS	14.44886364	THS	14.46306818	HBR	14.70454545
18	RSC	14.94602273	RSC	14.97159091	LAS	15.48295455
19	LAS	16.10795455	LAS	16.13636364	RSC	15.56107955
20	KNR	16.19602273	KNR	16.21590909	KNR	16.00568182
21	XGBoost	16.25284091	XGBoost	16.26136364	XGBoost	16.63068182
22	DTR	17.75568182	DTR	17.76988636	SVR	17.07386364
	**MAE**		**MAPE**		$R^{2}$	
	**Method**	**F-Rank**	**Method**	**F-Rank**	**Method**	**F-Rank**
1	LSTM	3.900568182	LSTM	4.022727273	LSTM	22.47443182
2	LSTM_2	4.340909091	LSTM_2	4.514204545	LSTM_2	22.20454545
3	LSTM_3	7.414772727	LSTM_3	7.400568182	LSTM_3	18.99715909
4	MLP	9.636363636	MLP	9.769886364	OMP	17.66903409
5	OMP	11.16619318	GB	11.14772727	MLP	17.59090909
6	GB	11.26136364	OMP	11.49857955	ARD	17.38636364
7	ARD	11.66761364	LGBM	11.84659091	LA	17.03267045
8	LA	11.88210227	ARD	11.94602273	LNR	16.73011364
9	LGBM	12.06818182	LA	12.14914773	RDG	16.65056818
10	LNR	12.25568182	LNR	12.53409091	GB	15.98579545
11	RDG	12.44602273	RDG	12.67045455	LGBM	15.40340909
12	CBR	12.99715909	CBR	12.95738636	ET	14.50568182
13	ET	13.21306818	ET	12.97159091	HBR	14.29545455
14	ABR	13.60795455	ABR	13.26704545	CBR	14.26704545
15	RF	13.73295455	RF	13.31534091	ABR	13.84943182
16	HBR	14.91477273	KNR	15.00852273	RF	13.82954545
17	KNR	15.39204545	HBR	15.18465909	THS	13.51136364
18	THS	15.39204545	XGBoost	15.34943182	RSC	13.13068182
19	XGBoost	15.57954545	THS	15.59090909	KNR	11.96590909
20	RSC	15.96875	SVR	16.13636364	XGBoost	11.94034091
21	SVR	16.26420455	RSC	16.21590909	LAS	11.90625
22	LAS	17.42613636	LAS	17.22727273	SVR	10.32102273

**Table A7:** Methods’ Friedman rankings (shift = 14).

	MSE		RMSE		RMSLE	
	**Method**	**F-Rank**	**Method**	**F-Rank**	**Method**	**F-Rank**
1	LSTM	5.488636364	LSTM	5.363636364	LSTM	4.946022727
2	LSTM_2	5.721590909	LSTM_2	5.588068182	LSTM_2	5.113636364
3	OMP	8.673295455	LSTM_3	8.443181818	LSTM_3	8.048295455
4	LSTM_3	8.678977273	OMP	8.701704545	MLP	9.488636364
5	ARD	9.116477273	ARD	9.15625	OMP	9.53125
6	LA	10.06107955	LA	10.09232955	ARD	9.840909091
7	MLP	10.18465909	MLP	10.20170455	LA	10.64914773
8	LNR	10.20880682	LNR	10.24005682	LNR	10.890625
9	RDG	10.29829545	RDG	10.32954545	RDG	11.01988636
10	HBR	11.86363636	HBR	11.88920455	HBR	12.58522727
11	ABR	13.26420455	ABR	13.29829545	ABR	13.15909091
12	RSC	13.46306818	RSC	13.48579545	THS	13.72301136
13	THS	13.53693182	THS	13.55113636	LAS	13.75
14	GB	13.98011364	GB	13.99431818	RSC	13.78267045
15	LAS	14.42329545	LAS	14.47443182	GB	14.16761364
16	LGBM	15.03977273	LGBM	15.06534091	LGBM	15.36079545
17	RF	16.18465909	RF	16.20170455	ET	16.46022727
18	ET	16.20170455	CBR	16.21590909	CBR	16.54261364
19	CBR	16.20454545	ET	16.22443182	RF	16.5625
20	KNR	16.54545455	KNR	16.57102273	KNR	16.61079545
21	SVR	17.40340909	SVR	17.41761364	SVR	17.04829545
22	XGBoost	17.73011364	XGBoost	17.75	ELN	17.54261364
	**MAE**		**MAPE**		$R^{2}$	
	**Method**	**F-Rank**	**Method**	**F-Rank**	**Method**	**F-Rank**
1	LSTM	5.539772727	LSTM	5.15625	LSTM	20.69318182
2	LSTM_2	5.732954545	LSTM_2	5.321022727	LSTM_2	20.46875
3	LSTM_3	8.784090909	LSTM_3	8.457386364	OMP	19.64204545
4	OMP	8.877840909	MLP	9.170454545	ARD	19.16193182
5	ARD	9.176136364	OMP	9.363636364	LA	18.21164773
6	MLP	9.295454545	ARD	9.653409091	LNR	18.06392045
7	LA	10.08664773	LA	10.43892045	MLP	18.04829545
8	LNR	10.27414773	LNR	10.640625	RDG	17.97159091
9	RDG	10.41761364	RDG	10.81818182	LSTM_3	17.69602273
10	HBR	11.94318182	HBR	12.39488636	HBR	16.48295455
11	ABR	13.18465909	ABR	12.83806818	ABR	14.87215909
12	THS	13.73863636	GB	13.61931818	RSC	14.76704545
13	GB	13.76988636	LGBM	14.16477273	THS	14.5
14	RSC	13.82102273	RSC	14.24431818	GB	14.24715909
15	LGBM	14.39204545	THS	14.28409091	LAS	13.97727273
16	CBR	15.61363636	CBR	15.29545455	LGBM	13.19034091
17	ET	15.76704545	ET	15.42613636	RF	12.11363636
18	RF	15.84090909	RF	15.44886364	ET	12.07670455
19	LAS	15.97159091	KNR	15.81534091	CBR	12.01704545
20	KNR	15.97443182	LAS	16.21875	KNR	11.71022727
21	SVR	16.46022727	SVR	16.35795455	SVR	10.80965909
22	XGBoost	17.72159091	XGBoost	17.42045455	XGBoost	10.55397727
